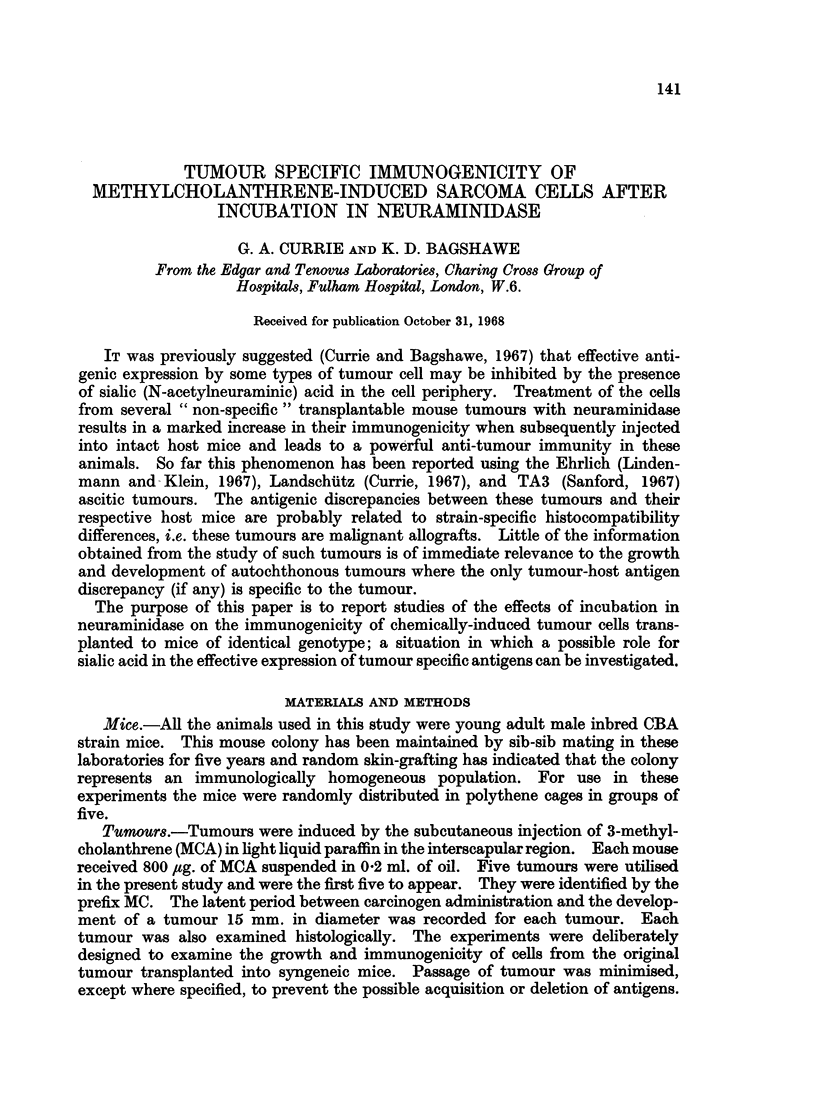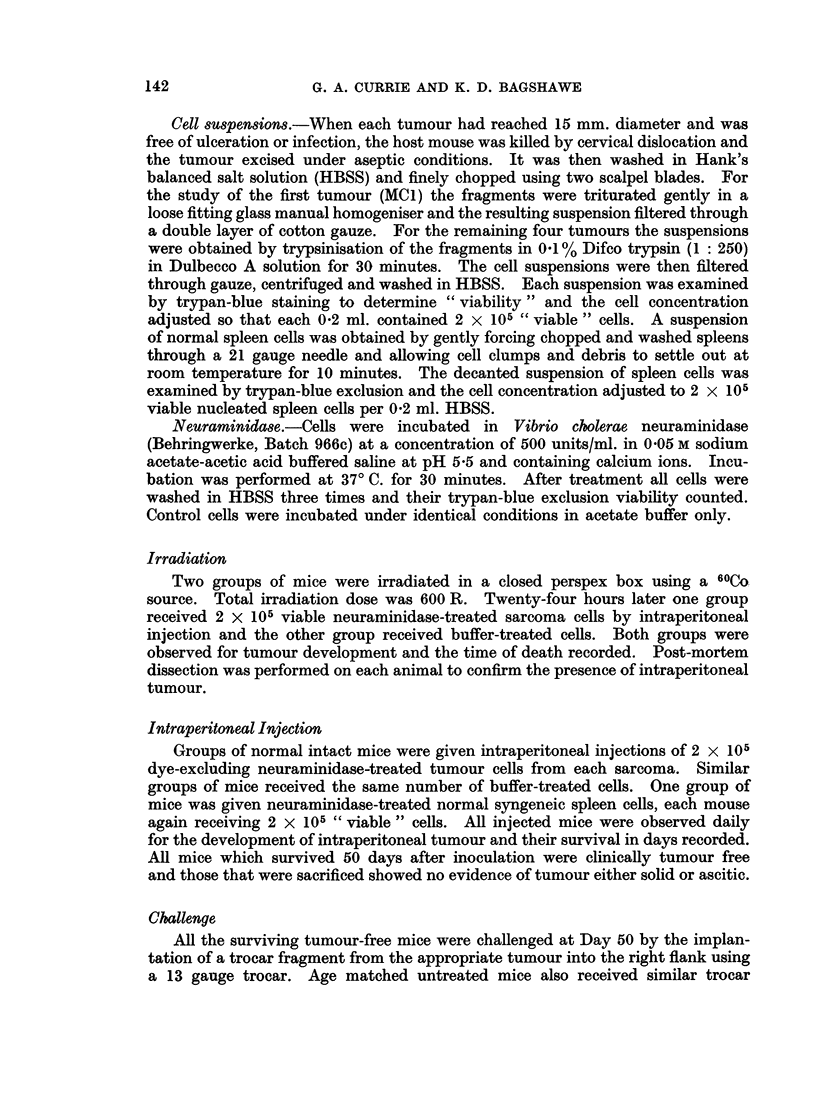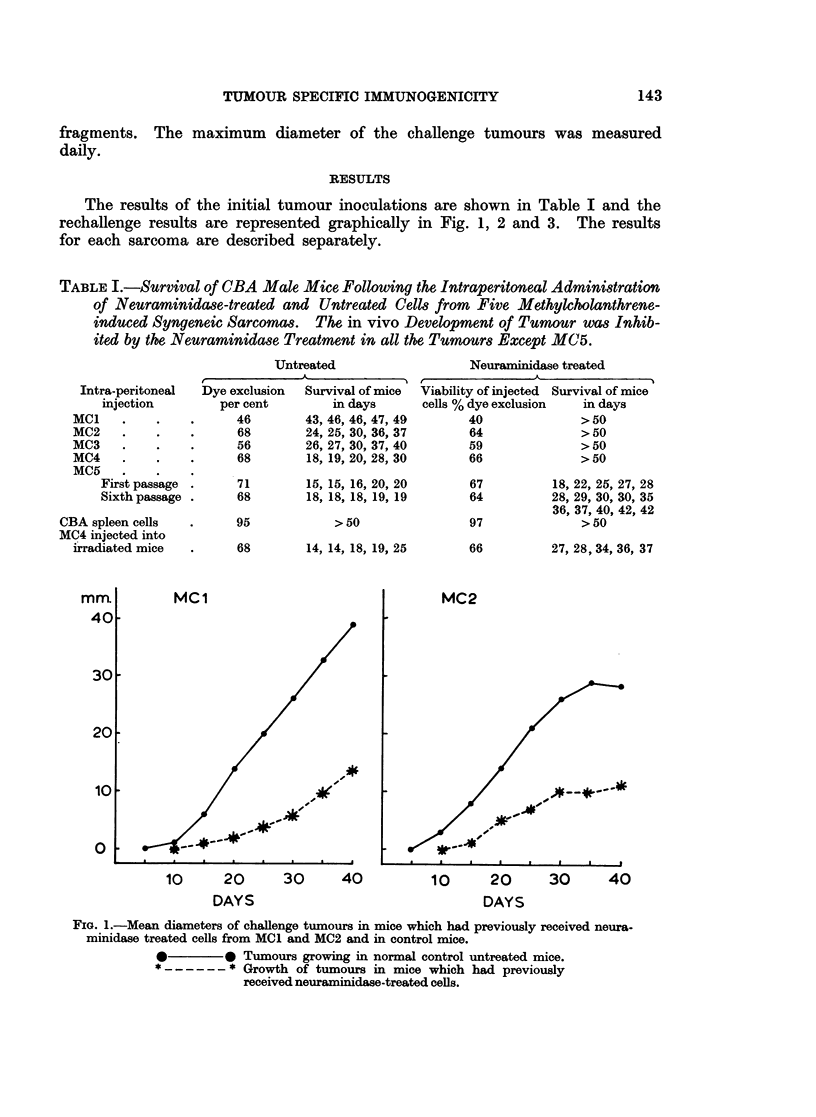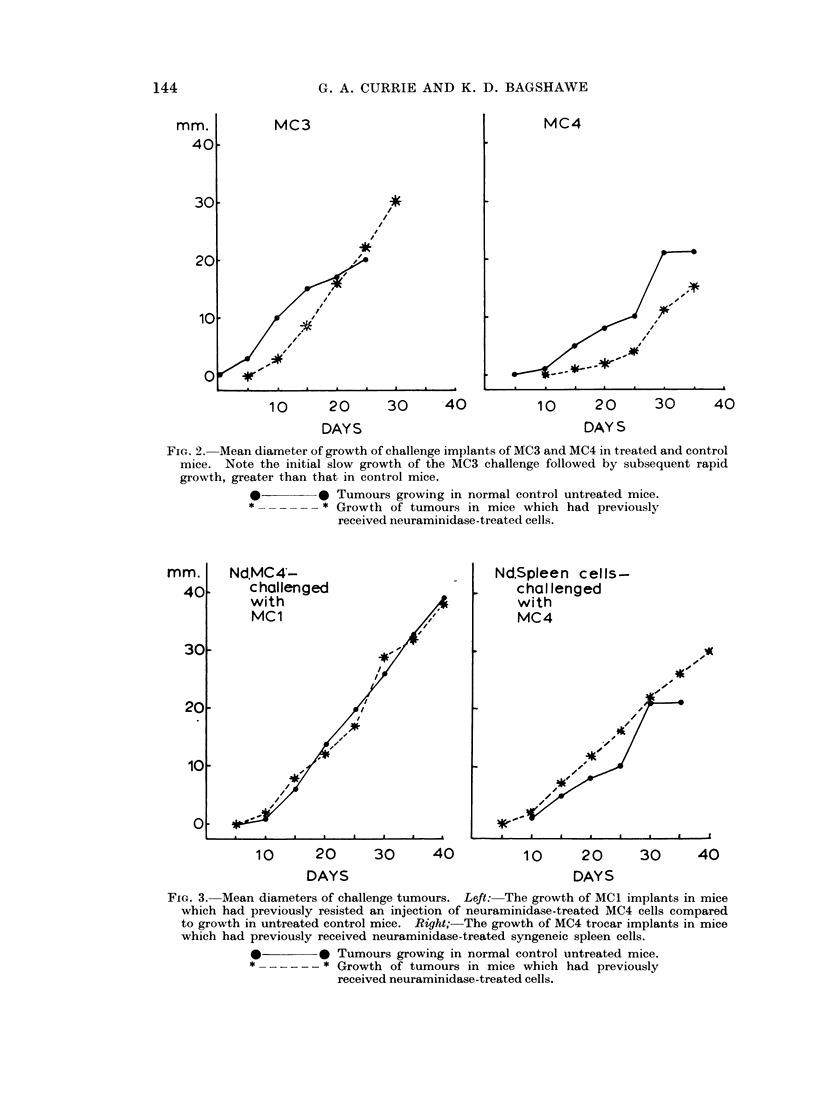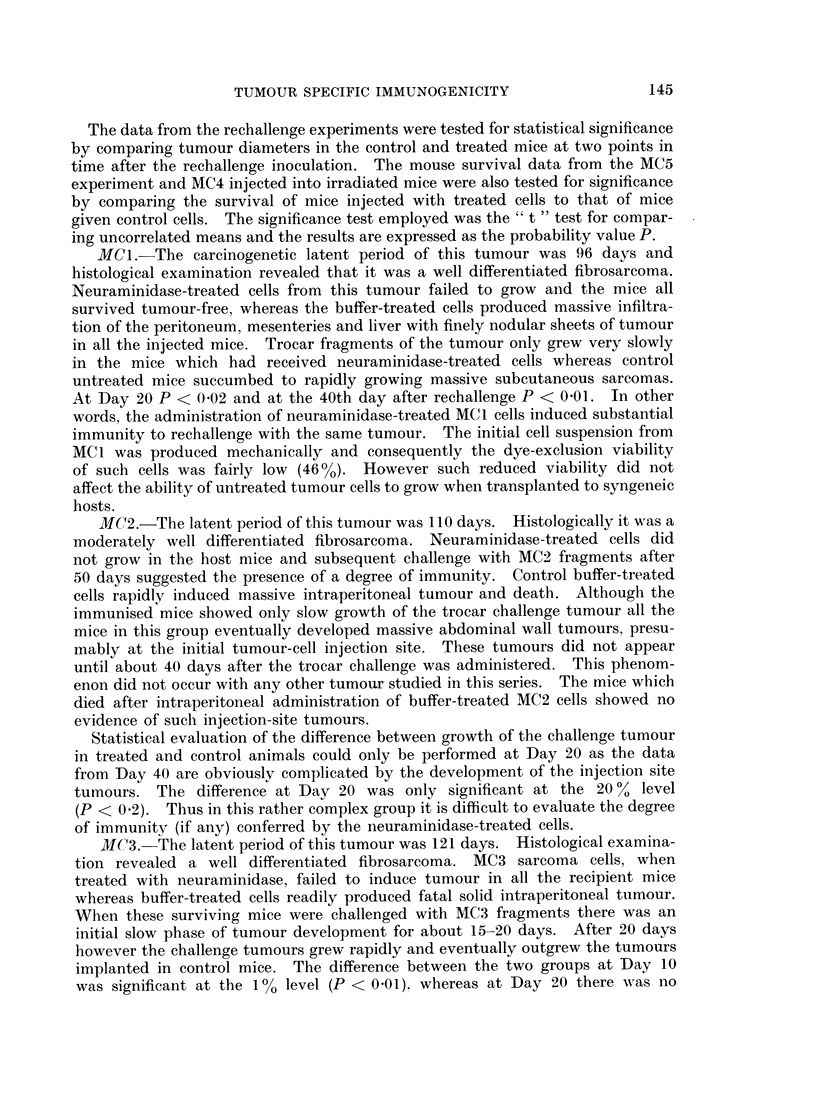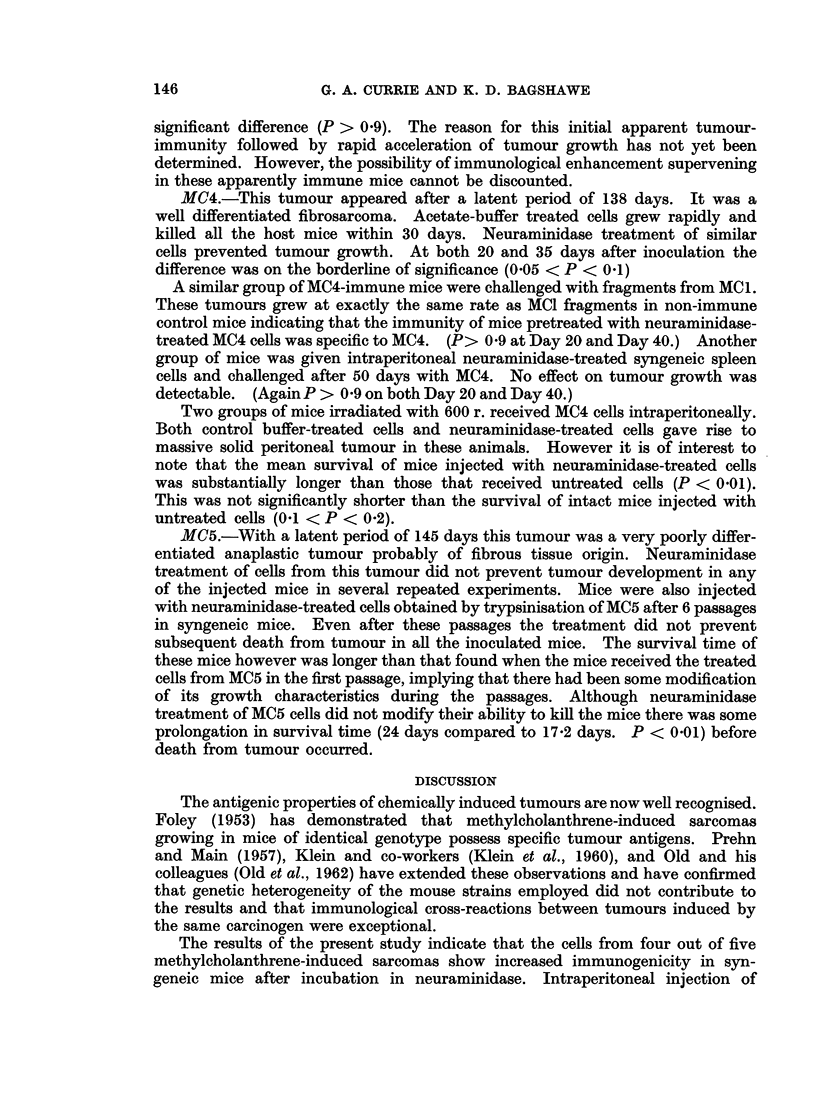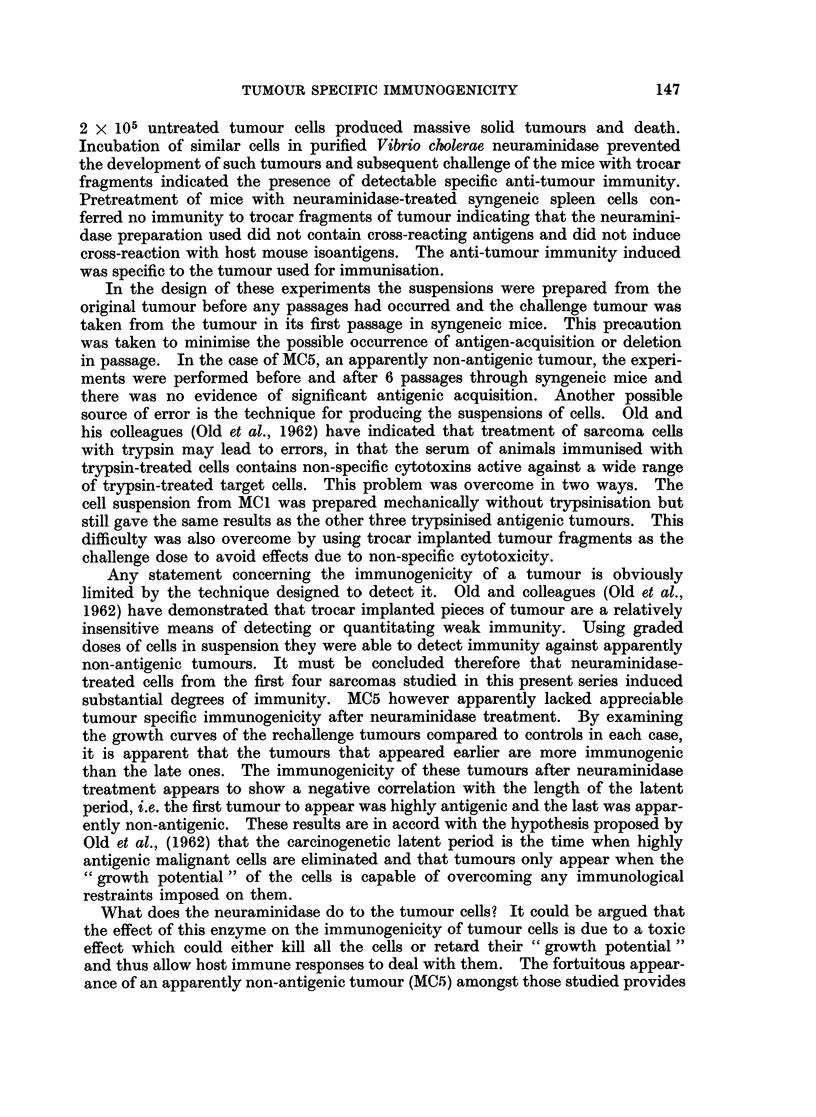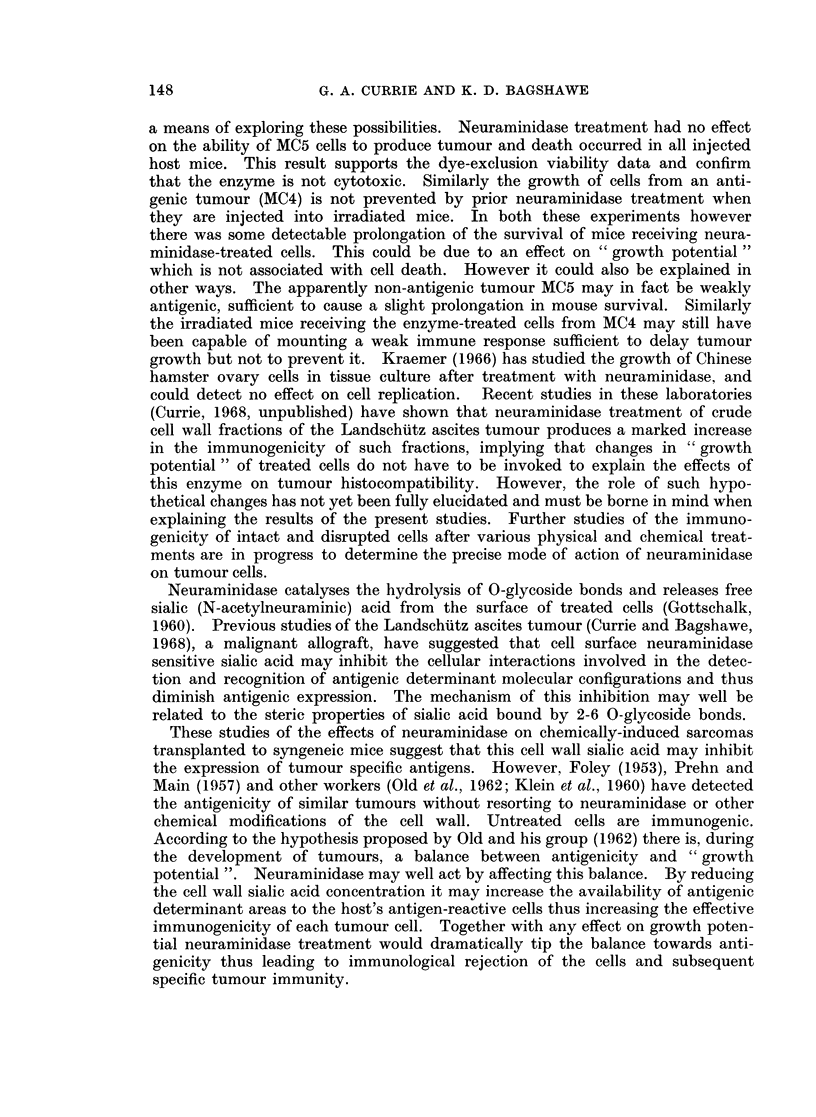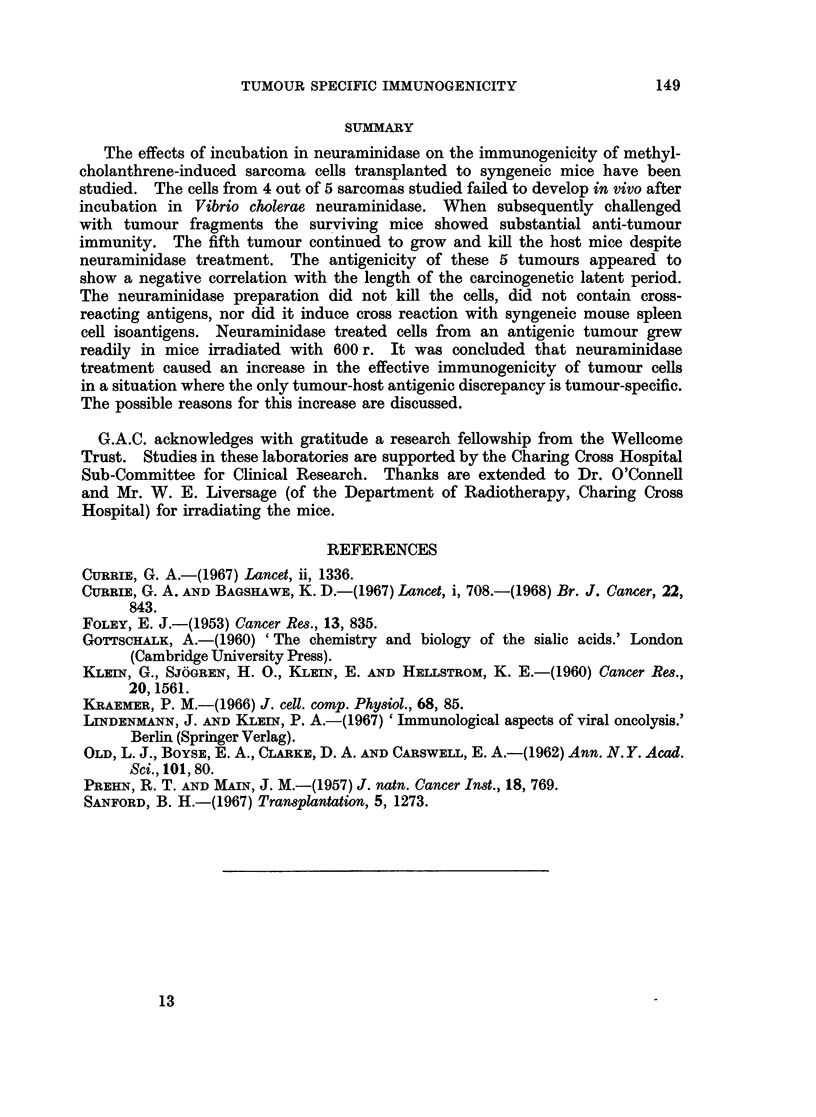# Tumour specific immunogenicity of methylcholanthrene-induced sarcoma cells after incubation in neuraminidase.

**DOI:** 10.1038/bjc.1969.21

**Published:** 1969-03

**Authors:** G. A. Currie, K. D. Bagshawe


### TUMOUR SPECIFIC IMMUNOGENICITY OF METHYLCHOLANTHRENE-INDUCED SARCOMA CELLS AFTER INCUBATION IN NEURAMINIDASE

G. A. CURRIE AND K. D. BAGSHAWE

*From the Edgar and Tenovus Laboratories, Charing Cross Group of Hospitals, Fulham Hospital, London, W.6.*

Received for publication October 31, 1968

It was previously suggested (Currie and Bagshawe, 1967) that effective antigenic expression by some types of tumour cell may be inhibited by the presence of sialic (N-acetylneuraminic) acid in the cell periphery. Treatment of the cells from several " non-specific " transplantable mouse tumours with neuraminidase results in a marked increase in their immunogenicity when subsequently injected into intact host mice and leads to a powerful anti-tumour immunity in these animals. So far this phenomenon has been reported using the Ehrlich (Lindenmann and Klein, 1967), Landschütz (Currie, 1967), and TA3 (Sanford, 1967) ascitic tumours. The antigenic discrepancies between these tumours and their respective host mice are probably related to strain-specific histocompatibility differences, *i.e.* these tumours are malignant allografts. Little of the information obtained from the study of such tumours is of immediate relevance to the growth and development of autochthonous tumours where the only tumour-host antigen discrepancy (if any) is specific to the tumour.

The purpose of this paper is to report studies of the effects of incubation in neuraminidase on the immunogenicity of chemically-induced tumour cells transplanted to mice of identical genotype; a situation in which a possible role for sialic acid in the effective expression of tumour specific antigens can be investigated.

### MATERIALS AND METHODS

*Mice.*—All the animals used in this study were young adult male inbred CBA strain mice. This mouse colony has been maintained by sib-sib mating in these laboratories for five years and random skin-grafting has indicated that the colony represents an immunologically homogeneous population. For use in these experiments the mice were randomly distributed in polythene cages in groups of five.

*Tumours.*—Tumours were induced by the subcutaneous injection of 3-methylcholanthrene (MCA) in light liquid paraffin in the interscapular region. Each mouse received 800 $\mu$g. of MCA suspended in 0·2 ml. of oil. Five tumours were utilised in the present study and were the first five to appear. They were identified by the prefix MC. The latent period between carcinogen administration and the development of a tumour 15 mm. in diameter was recorded for each tumour. Each tumour was also examined histologically. The experiments were deliberately designed to examine the growth and immunogenicity of cells from the original tumour transplanted into syngeneic mice. Passage of tumour was minimised, except where specified, to prevent the possible acquisition or deletion of antigens.

*Cell suspensions.*—When each tumour had reached 15 mm. diameter and was free of ulceration or infection, the host mouse was killed by cervical dislocation and the tumour excised under aseptic conditions. It was then washed in Hank's balanced salt solution (HBSS) and finely chopped using two scalpel blades. For the study of the first tumour (MC1) the fragments were triturated gently in a loose fitting glass manual homogeniser and the resulting suspension filtered through a double layer of cotton gauze. For the remaining four tumours the suspensions were obtained by trypsinisation of the fragments in 0·1% Difco trypsin (1 : 250) in Dulbecco A solution for 30 minutes. The cell suspensions were then filtered through gauze, centrifuged and washed in HBSS. Each suspension was examined by trypan-blue staining to determine " viability " and the cell concentration adjusted so that each 0·2 ml. contained $2 \times 10^5$ " viable " cells. A suspension of normal spleen cells was obtained by gently forcing chopped and washed spleens through a 21 gauge needle and allowing cell clumps and debris to settle out at room temperature for 10 minutes. The decanted suspension of spleen cells was examined by trypan-blue exclusion and the cell concentration adjusted to $2 \times 10^5$ viable nucleated spleen cells per 0·2 ml. HBSS.

*Neuraminidase.*—Cells were incubated in *Vibrio cholerae* neuraminidase (Behringwerke, Batch 966c) at a concentration of 500 units/ml. in 0·05 M sodium acetate-acetic acid buffered saline at pH 5·5 and containing calcium ions. Incubation was performed at 37° C. for 30 minutes. After treatment all cells were washed in HBSS three times and their trypan-blue exclusion viability counted. Control cells were incubated under identical conditions in acetate buffer only.

### *Irradiation*

Two groups of mice were irradiated in a closed perspex box using a $^{60}Co$ source. Total irradiation dose was 600 R. Twenty-four hours later one group received $2 \times 10^5$ viable neuraminidase-treated sarcoma cells by intraperitoneal injection and the other group received buffer-treated cells. Both groups were observed for tumour development and the time of death recorded. Post-mortem dissection was performed on each animal to confirm the presence of intraperitoneal tumour.

### *Intraperitoneal Injection*

Groups of normal intact mice were given intraperitoneal injections of $2 \times 10^5$ dye-excluding neuraminidase-treated tumour cells from each sarcoma. Similar groups of mice received the same number of buffer-treated cells. One group of mice was given neuraminidase-treated normal syngeneic spleen cells, each mouse again receiving $2 \times 10^5$ " viable " cells. All injected mice were observed daily for the development of intraperitoneal tumour and their survival in days recorded. All mice which survived 50 days after inoculation were clinically tumour free and those that were sacrificed showed no evidence of tumour either solid or ascitic.

### *Challenge*

All the surviving tumour-free mice were challenged at Day 50 by the implantation of a trocar fragment from the appropriate tumour into the right flank using a 13 gauge trocar. Age matched untreated mice also received similar trocar fragments. The maximum diameter of the challenge tumours was measured daily.

### RESULTS

The results of the initial tumour inoculations are shown in Table I and the rechallenge results are represented graphically in Fig. 1, 2 and 3. The results for each sarcoma are described separately.

TABLE I.—*Survival of CBA Male Mice Following the Intraperitoneal Administration of Neuraminidase-treated and Untreated Cells from Five Methylcholanthrene-induced Syngeneic Sarcomas. The* in vivo *Development of Tumour was Inhibited by the Neuraminidase Treatment in all the Tumours Except MC5.*

| | Untreated | | Neuraminidase treated | |
|---|---|---|---|---|
| Intra-peritoneal injection | Dye exclusion per cent | Survival of mice in days | Viability of injected cells % dye exclusion | Survival of mice in days |
| MC1 | 46 | 43, 46, 46, 47, 49 | 40 | > 50 |
| MC2 | 68 | 24, 25, 30, 36, 37 | 64 | > 50 |
| MC3 | 56 | 26, 27, 30, 37, 40 | 59 | > 50 |
| MC4 | 68 | 18, 19, 20, 28, 30 | 66 | > 50 |
| MC5 | | | | |
| First passage | 71 | 15, 15, 16, 20, 20 | 67 | 18, 22, 25, 27, 28 |
| Sixth passage | 68 | 18, 18, 18, 19, 19 | 64 | 28, 29, 30, 30, 35 36, 37, 40, 42, 42 |
| CBA spleen cells | 95 | > 50 | 97 | > 50 |
| MC4 injected into irradiated mice | 68 | 14, 14, 18, 19, 25 | 66 | 27, 28, 34, 36, 37 |

FIG. 1.—Mean diameters of challenge tumours in mice which had previously received neuraminidase treated cells from MC1 and MC2 and in control mice.

●———● Tumours growing in normal control untreated mice.
*- - - - - -* Growth of tumours in mice which had previously received neuraminidase-treated cells.

FIG. 2.—Mean diameter of growth of challenge implants of MC3 and MC4 in treated and control mice. Note the initial slow growth of the MC3 challenge followed by subsequent rapid growth, greater than that in control mice.

●———● Tumours growing in normal control untreated mice.
* - - - - - - * Growth of tumours in mice which had previously received neuraminidase-treated cells.

FIG. 3.—Mean diameters of challenge tumours. *Left*:—The growth of MC1 implants in mice which had previously resisted an injection of neuraminidase-treated MC4 cells compared to growth in untreated control mice. *Right*;—The growth of MC4 trocar implants in mice which had previously received neuraminidase-treated syngeneic spleen cells.

●———● Tumours growing in normal control untreated mice.
* - - - - - - * Growth of tumours in mice which had previously received neuraminidase-treated cells.

The data from the rechallenge experiments were tested for statistical significance by comparing tumour diameters in the control and treated mice at two points in time after the rechallenge inoculation. The mouse survival data from the MC5 experiment and MC4 injected into irradiated mice were also tested for significance by comparing the survival of mice injected with treated cells to that of mice given control cells. The significance test employed was the " t " test for comparing uncorrelated means and the results are expressed as the probability value $P$.

*MC*1.—The carcinogenetic latent period of this tumour was 96 days and histological examination revealed that it was a well differentiated fibrosarcoma. Neuraminidase-treated cells from this tumour failed to grow and the mice all survived tumour-free, whereas the buffer-treated cells produced massive infiltration of the peritoneum, mesenteries and liver with finely nodular sheets of tumour in all the injected mice. Trocar fragments of the tumour only grew very slowly in the mice which had received neuraminidase-treated cells whereas control untreated mice succumbed to rapidly growing massive subcutaneous sarcomas. At Day 20 $P < 0{\cdot}02$ and at the 40th day after rechallenge $P < 0{\cdot}01$. In other words, the administration of neuraminidase-treated MC1 cells induced substantial immunity to rechallenge with the same tumour. The initial cell suspension from MC1 was produced mechanically and consequently the dye-exclusion viability of such cells was fairly low (46%). However such reduced viability did not affect the ability of untreated tumour cells to grow when transplanted to syngeneic hosts.

*MC*2.—The latent period of this tumour was 110 days. Histologically it was a moderately well differentiated fibrosarcoma. Neuraminidase-treated cells did not grow in the host mice and subsequent challenge with MC2 fragments after 50 days suggested the presence of a degree of immunity. Control buffer-treated cells rapidly induced massive intraperitoneal tumour and death. Although the immunised mice showed only slow growth of the trocar challenge tumour all the mice in this group eventually developed massive abdominal wall tumours, presumably at the initial tumour-cell injection site. These tumours did not appear until about 40 days after the trocar challenge was administered. This phenomenon did not occur with any other tumour studied in this series. The mice which died after intraperitoneal administration of buffer-treated MC2 cells showed no evidence of such injection-site tumours.

Statistical evaluation of the difference between growth of the challenge tumour in treated and control animals could only be performed at Day 20 as the data from Day 40 are obviously complicated by the development of the injection site tumours. The difference at Day 20 was only significant at the 20% level ($P < 0{\cdot}2$). Thus in this rather complex group it is difficult to evaluate the degree of immunity (if any) conferred by the neuraminidase-treated cells.

*MC*3.—The latent period of this tumour was 121 days. Histological examination revealed a well differentiated fibrosarcoma. MC3 sarcoma cells, when treated with neuraminidase, failed to induce tumour in all the recipient mice whereas buffer-treated cells readily produced fatal solid intraperitoneal tumour. When these surviving mice were challenged with MC3 fragments there was an initial slow phase of tumour development for about 15–20 days. After 20 days however the challenge tumours grew rapidly and eventually outgrew the tumours implanted in control mice. The difference between the two groups at Day 10 was significant at the 1% level ($P < 0{\cdot}01$), whereas at Day 20 there was no significant difference ($P > 0 \cdot 9$). The reason for this initial apparent tumour-immunity followed by rapid acceleration of tumour growth has not yet been determined. However, the possibility of immunological enhancement supervening in these apparently immune mice cannot be discounted.

*MC*4.—This tumour appeared after a latent period of 138 days. It was a well differentiated fibrosarcoma. Acetate-buffer treated cells grew rapidly and killed all the host mice within 30 days. Neuraminidase treatment of similar cells prevented tumour growth. At both 20 and 35 days after inoculation the difference was on the borderline of significance ($0 \cdot 05 < P < 0 \cdot 1$)

A similar group of MC4-immune mice were challenged with fragments from MC1. These tumours grew at exactly the same rate as MCl fragments in non-immune control mice indicating that the immunity of mice pretreated with neuraminidase-treated MC4 cells was specific to MC4. ($P > 0 \cdot 9$ at Day 20 and Day 40.) Another group of mice was given intraperitoneal neuraminidase-treated syngeneic spleen cells and challenged after 50 days with MC4. No effect on tumour growth was detectable. (Again $P > 0 \cdot 9$ on both Day 20 and Day 40.)

Two groups of mice irradiated with 600 r. received MC4 cells intraperitoneally. Both control buffer-treated cells and neuraminidase-treated cells gave rise to massive solid peritoneal tumour in these animals. However it is of interest to note that the mean survival of mice injected with neuraminidase-treated cells was substantially longer than those that received untreated cells ($P < 0 \cdot 01$). This was not significantly shorter than the survival of intact mice injected with untreated cells ($0 \cdot 1 < P < 0 \cdot 2$).

*MC*5.—With a latent period of 145 days this tumour was a very poorly differentiated anaplastic tumour probably of fibrous tissue origin. Neuraminidase treatment of cells from this tumour did not prevent tumour development in any of the injected mice in several repeated experiments. Mice were also injected with neuraminidase-treated cells obtained by trypsinisation of MC5 after 6 passages in syngeneic mice. Even after these passages the treatment did not prevent subsequent death from tumour in all the inoculated mice. The survival time of these mice however was longer than that found when the mice received the treated cells from MC5 in the first passage, implying that there had been some modification of its growth characteristics during the passages. Although neuraminidase treatment of MC5 cells did not modify their ability to kill the mice there was some prolongation in survival time (24 days compared to 17·2 days. $P < 0 \cdot 01$) before death from tumour occurred.

### DISCUSSION

The antigenic properties of chemically induced tumours are now well recognised. Foley (1953) has demonstrated that methylcholanthrene-induced sarcomas growing in mice of identical genotype possess specific tumour antigens. Prehn and Main (1957), Klein and co-workers (Klein *et al.*, 1960), and Old and his colleagues (Old *et al.*, 1962) have extended these observations and have confirmed that genetic heterogeneity of the mouse strains employed did not contribute to the results and that immunological cross-reactions between tumours induced by the same carcinogen were exceptional.

The results of the present study indicate that the cells from four out of five methylcholanthrene-induced sarcomas show increased immunogenicity in syngeneic mice after incubation in neuraminidase. Intraperitoneal injection of

$2 \times 10^5$ untreated tumour cells produced massive solid tumours and death. Incubation of similar cells in purified *Vibrio cholerae* neuraminidase prevented the development of such tumours and subsequent challenge of the mice with trocar fragments indicated the presence of detectable specific anti-tumour immunity. Pretreatment of mice with neuraminidase-treated syngeneic spleen cells conferred no immunity to trocar fragments of tumour indicating that the neuraminidase preparation used did not contain cross-reacting antigens and did not induce cross-reaction with host mouse isoantigens. The anti-tumour immunity induced was specific to the tumour used for immunisation.

In the design of these experiments the suspensions were prepared from the original tumour before any passages had occurred and the challenge tumour was taken from the tumour in its first passage in syngeneic mice. This precaution was taken to minimise the possible occurrence of antigen-acquisition or deletion in passage. In the case of MC5, an apparently non-antigenic tumour, the experiments were performed before and after 6 passages through syngeneic mice and there was no evidence of significant antigenic acquisition. Another possible source of error is the technique for producing the suspensions of cells. Old and his colleagues (Old *et al.*, 1962) have indicated that treatment of sarcoma cells with trypsin may lead to errors, in that the serum of animals immunised with trypsin-treated cells contains non-specific cytotoxins active against a wide range of trypsin-treated target cells. This problem was overcome in two ways. The cell suspension from MC1 was prepared mechanically without trypsinisation but still gave the same results as the other three trypsinised antigenic tumours. This difficulty was also overcome by using trocar implanted tumour fragments as the challenge dose to avoid effects due to non-specific cytotoxicity.

Any statement concerning the immunogenicity of a tumour is obviously limited by the technique designed to detect it. Old and colleagues (Old *et al.*, 1962) have demonstrated that trocar implanted pieces of tumour are a relatively insensitive means of detecting or quantitating weak immunity. Using graded doses of cells in suspension they were able to detect immunity against apparently non-antigenic tumours. It must be concluded therefore that neuraminidase-treated cells from the first four sarcomas studied in this present series induced substantial degrees of immunity. MC5 however apparently lacked appreciable tumour specific immunogenicity after neuraminidase treatment. By examining the growth curves of the rechallenge tumours compared to controls in each case, it is apparent that the tumours that appeared earlier are more immunogenic than the late ones. The immunogenicity of these tumours after neuraminidase treatment appears to show a negative correlation with the length of the latent period, *i.e.* the first tumour to appear was highly antigenic and the last was apparently non-antigenic. These results are in accord with the hypothesis proposed by Old *et al.*, (1962) that the carcinogenetic latent period is the time when highly antigenic malignant cells are eliminated and that tumours only appear when the "growth potential" of the cells is capable of overcoming any immunological restraints imposed on them.

What does the neuraminidase do to the tumour cells? It could be argued that the effect of this enzyme on the immunogenicity of tumour cells is due to a toxic effect which could either kill all the cells or retard their "growth potential" and thus allow host immune responses to deal with them. The fortuitous appearance of an apparently non-antigenic tumour (MC5) amongst those studied provides a means of exploring these possibilities. Neuraminidase treatment had no effect on the ability of MC5 cells to produce tumour and death occurred in all injected host mice. This result supports the dye-exclusion viability data and confirm that the enzyme is not cytotoxic. Similarly the growth of cells from an antigenic tumour (MC4) is not prevented by prior neuraminidase treatment when they are injected into irradiated mice. In both these experiments however there was some detectable prolongation of the survival of mice receiving neuraminidase-treated cells. This could be due to an effect on " growth potential " which is not associated with cell death. However it could also be explained in other ways. The apparently non-antigenic tumour MC5 may in fact be weakly antigenic, sufficient to cause a slight prolongation in mouse survival. Similarly the irradiated mice receiving the enzyme-treated cells from MC4 may still have been capable of mounting a weak immune response sufficient to delay tumour growth but not to prevent it. Kraemer (1966) has studied the growth of Chinese hamster ovary cells in tissue culture after treatment with neuraminidase, and could detect no effect on cell replication. Recent studies in these laboratories (Currie, 1968, unpublished) have shown that neuraminidase treatment of crude cell wall fractions of the Landschütz ascites tumour produces a marked increase in the immunogenicity of such fractions, implying that changes in " growth potential " of treated cells do not have to be invoked to explain the effects of this enzyme on tumour histocompatibility. However, the role of such hypothetical changes has not yet been fully elucidated and must be borne in mind when explaining the results of the present studies. Further studies of the immunogenicity of intact and disrupted cells after various physical and chemical treatments are in progress to determine the precise mode of action of neuraminidase on tumour cells.

Neuraminidase catalyses the hydrolysis of O-glycoside bonds and releases free sialic (N-acetylneuraminic) acid from the surface of treated cells (Gottschalk, 1960). Previous studies of the Landschütz ascites tumour (Currie and Bagshawe, 1968), a malignant allograft, have suggested that cell surface neuraminidase sensitive sialic acid may inhibit the cellular interactions involved in the detection and recognition of antigenic determinant molecular configurations and thus diminish antigenic expression. The mechanism of this inhibition may well be related to the steric properties of sialic acid bound by 2-6 O-glycoside bonds.

These studies of the effects of neuraminidase on chemically-induced sarcomas transplanted to syngeneic mice suggest that this cell wall sialic acid may inhibit the expression of tumour specific antigens. However, Foley (1953), Prehn and Main (1957) and other workers (Old *et al.*, 1962; Klein *et al.*, 1960) have detected the antigenicity of similar tumours without resorting to neuraminidase or other chemical modifications of the cell wall. Untreated cells are immunogenic. According to the hypothesis proposed by Old and his group (1962) there is, during the development of tumours, a balance between antigenicity and " growth potential ". Neuraminidase may well act by affecting this balance. By reducing the cell wall sialic acid concentration it may increase the availability of antigenic determinant areas to the host's antigen-reactive cells thus increasing the effective immunogenicity of each tumour cell. Together with any effect on growth potential neuraminidase treatment would dramatically tip the balance towards antigenicity thus leading to immunological rejection of the cells and subsequent specific tumour immunity.

SUMMARY

The effects of incubation in neuraminidase on the immunogenicity of methylcholanthrene-induced sarcoma cells transplanted to syngeneic mice have been studied. The cells from 4 out of 5 sarcomas studied failed to develop *in vivo* after incubation in *Vibrio cholerae* neuraminidase. When subsequently challenged with tumour fragments the surviving mice showed substantial anti-tumour immunity. The fifth tumour continued to grow and kill the host mice despite neuraminidase treatment. The antigenicity of these 5 tumours appeared to show a negative correlation with the length of the carcinogenetic latent period. The neuraminidase preparation did not kill the cells, did not contain cross-reacting antigens, nor did it induce cross reaction with syngeneic mouse spleen cell isoantigens. Neuraminidase treated cells from an antigenic tumour grew readily in mice irradiated with 600 r. It was concluded that neuraminidase treatment caused an increase in the effective immunogenicity of tumour cells in a situation where the only tumour-host antigenic discrepancy is tumour-specific. The possible reasons for this increase are discussed.

G.A.C. acknowledges with gratitude a research fellowship from the Wellcome Trust. Studies in these laboratories are supported by the Charing Cross Hospital Sub-Committee for Clinical Research. Thanks are extended to Dr. O'Connell and Mr. W. E. Liversage (of the Department of Radiotherapy, Charing Cross Hospital) for irradiating the mice.

### REFERENCES

Currie, G. A.—(1967) *Lancet*, ii, 1336.

Currie, G. A. and Bagshawe, K. D.—(1967) *Lancet*, i, 708.—(1968) *Br. J. Cancer*, **22**, 843.

Foley, E. J.—(1953) *Cancer Res.*, **13**, 835.

Gottschalk, A.—(1960) 'The chemistry and biology of the sialic acids.' London (Cambridge University Press).

Klein, G., Sjögren, H. O., Klein, E. and Hellstrom, K. E.—(1960) *Cancer Res.*, **20**, 1561.

Kraemer, P. M.—(1966) *J. cell. comp. Physiol.*, **68**, 85.

Lindenmann, J. and Klein, P. A.—(1967) 'Immunological aspects of viral oncolysis.' Berlin (Springer Verlag).

Old, L. J., Boyse, E. A., Clarke, D. A. and Carswell, E. A.—(1962) *Ann. N.Y. Acad. Sci.*, **101**, 80.

Prehn, R. T. and Main, J. M.—(1957) *J. natn. Cancer Inst.*, **18**, 769.

Sanford, B. H.—(1967) *Transplantation*, **5**, 1273.